# Accelerated vascular aging: Ethnic differences in basilar artery length and diameter, and its association with cardiovascular risk factors and cerebral small vessel disease

**DOI:** 10.3389/fcvm.2022.939680

**Published:** 2022-07-28

**Authors:** Carole H. Sudre, Stefano Moriconi, Rafael Rehwald, Lorna Smith, Therese Tillin, Josephine Barnes, David Atkinson, Sébastien Ourselin, Nish Chaturvedi, Alun D. Hughes, H. Rolf Jäger, M. Jorge Cardoso

**Affiliations:** ^1^MRC Unit for Lifelong Health and Ageing at UCL, Department of Population Science and Experimental Medicine, UCL Institute of Cardiovascular Science, University College London, London, United Kingdom; ^2^Department of Computer Science, Centre for Medical Image Computing, University College London, London, United Kingdom; ^3^School of Biomedical Engineering and Imaging Sciences, King's College London, London, United Kingdom; ^4^Department of Radiology, School of Clinical Medicine, University of Cambridge, Cambridge, United Kingdom; ^5^Neuroradiological Academic Unit, Department of Brain Repair and Rehabilitation, Queen Square Institute of Neurology, University College London, London, United Kingdom; ^6^Centre for Medical Imaging, Division of Medicine, University College London, London, United Kingdom; ^7^Dementia Research Centre, UCL Institute of Neurology, University College London, London, United Kingdom

**Keywords:** basilar artery (BA), white matter hyperintensities (WMH), aging, ethnicity, early vascular aging, cerebral small vessel disease

## Abstract

**Background and aims:**

Risk of stroke and dementia is markedly higher in people of South Asian and African Caribbean descent than white Europeans in the UK. This is unexplained by cardiovascular risk factors (CVRF). We hypothesized this might indicate accelerated early vascular aging (EVA) and that EVA might account for stronger associations between cerebral large artery characteristics and markers of small vessel disease.

**Methods:**

360 participants in a tri-ethnic population-based study (120 per ethnic group) underwent cerebral and vertebral MRI. Length and median diameter of the basilar artery (BA) were derived from Time of Flight images, while white matter hyperintensities (WMH) volumes were obtained from T1 and FLAIR images. Associations between BA characteristics and CVRF were assessed using multivariable linear regression. Partial correlation coefficients between WMH load and BA characteristics were calculated after adjustment for CVRF and other potential confounders.

**Results:**

BA diameter was strongly associated with age in South Asians (+11.3 μm/year 95% CI = [3.05; 19.62]; *p* = 0.008), with unconvincing relationships in African Caribbeans (3.4 μm/year [−5.26, 12.12]; *p* = 0.436) or Europeans (2.6 μm/year [−5.75, 10.87]; *p* = 0.543). BA length was associated with age in South Asians (+0.34 mm/year [0.02; 0.65]; *p* = 0.037) and African Caribbeans (+0.39 mm/year [0.12; 0.65]; *p* = 0.005) but not Europeans (+0.08 mm/year [−0.26; 0.41]; *p* = 0.653). BA diameter (rho = 0.210; *p* = 0.022) and length (rho = 0.261; *p* = 0.004) were associated with frontal WMH load in South Asians (persisting after multivariable adjustment for CVRF).

**Conclusions:**

Compared with Europeans, the basilar artery undergoes more accelerated EVA in South Asians and in African Caribbeans, albeit to a lesser extent. Such EVA may contribute to the higher burden of CSVD observed in South Asians and excess risk of stroke, vascular cognitive impairment and dementia observed in these ethnic groups.

## Introduction

People of South Asian and of Black African descent have a ~50% excess stroke risk compared with white Europeans in the UK (1) and are at higher risk of vascular cognitive impairment and dementia (2–4). Although there are ethnic differences in blood pressure (BP) and diabetes, these do not explain this elevated risk of stroke or cerebral small vessel disease (CSVD) in these ethnic minorities (5, 6) and a plausible explanation is that vascular aging is accelerated in these groups.

Early vascular aging (EVA) refers to an accelerated and dysregulated form of normal vascular aging that affects both macro and micro-vessels (7). Cardiovascular risk factors are associated with EVA through their effects on atherosclerosis and arteriosclerosis (8, 9), and identification of EVA in the cerebral circulation could potentially yield useful insights into ethnic differences in susceptibility to cerebrovascular disease.

Vessel dilatation and elongation are characteristic features of EVA: arteries are well known to elongate with age (10–12) and atherosclerosis is associated with compensatory dilation (6). *Ex vivo* analyses have indicated that increased arterial stiffness, dilation and elongation are linked through changes in biomechanical properties of arteries (13), possibly due to loss of elastic fibers (7). Indeed, modeling studies suggest a key and early role for axial stress in arterial remodeling in response to aging and elevated BP (14). EVA may be particular prominent in the posterior cerebral circulation, and the basilar artery is an ideal candidate for investigation of EVA (15). Formed by the union of the vertebral arteries, it is the main vessel providing blood supply to the posterior circulation of the brain, accounting for a quarter of total intra-cranial blood flow. Being a single vessel it obviates the need to examine and integrate findings bilaterally, as would be the case for the internal carotid arteries where lateralised compensatory effects may be observed (11). Aging is associated with a greater diameter and length of the basilar artery (10, 16) and increased basilar artery diameter has been shown to predict cardiovascular disease independent of other risk factors (16) and to be associated with a greater burden of white matter hyperintensities (WMH) (16, 17).

We therefore investigated the relationship between cardiovascular risk factors and basilar artery geometric characteristics as a measure of EVA across three ethnic groups. We then assessed the association of these risk factors with the presence of white matter hyperintensities as an indicator of small vessel disease (SVD). Finally, hypothesizing possible differences across ethnicities in the vascular aging mechanisms, we studied the association between basilar artery characteristics and SVD burden independent of classical cardiovascular risk factors.

## Methods

### Data availability

The data that support the findings of this study are available from the SABRE study (https://www.sabrestudy.org/) upon reasonable request.

### Participants

Participants were drawn from the SABRE (Southall and Brent revisited) cohort (18). This is a population-based tri-ethnic study of people of white European (EU), South Asian (SA) and African Caribbean (AC) descent. Ethnicity was agreed with the interviewer using self-report and country of birth information. According to the UK census of 2001, South Asian ethnicity refers to individuals with Indian, Pakistani or Bangladeshi ancestry. Ethnic minority groups were exclusively first-generation migrants. Of the 1,006 participants attending clinical assessment, 741 underwent vertebral MRI (310 EU, 247 SA, 175 AC, 9 other). The study received ethical approval from the National Research Ethics Service Committee, London-Fulham (14/LO/0108) and all participants provided written informed consent.

Participants showing one hypoplastic vertebral artery, terminating into the ipsilateral posterior inferior cerebellar artery without any rudimentary anastomosis with the contralateral vertebral artery, were excluded from the analysis (21 European, 22 South Asian and 40 African Caribbean). As image labeling is labor intensive, we selected the first consecutive 120 participants from each ethnic group for detailed analysis of the basilar artery. Images of all selected participants showed both vertebral arteries fusing into the basilar artery.

### Clinical data

Participants underwent all examinations on the same day as MRI. Participants were requested to consume no more than an early light breakfast prior to arrival at the clinic. Weight and height were measured using a Tanita Pro BC-418 Body Composition Analyser and a Seca 216 Stadiometer, respectively, and body mass index (BMI) was calculated. Questionnaires ascertained key lifestyle and demographic characteristics, past medical history (including doctor diagnosis of cardiovascular disease, hypertension and diabetes), and current medication. Blood pressure (BP) was measured in a seated position following the guidelines from 2013 of the European Society of Hypertension/European Society of Cardiology after 5 min of rest using an Omron MIT Elite Plus blood pressure monitor with an appropriate cuff size. The average of the last two available measurements from the left arm was taken unless the difference of measurement between arms was larger than 10 mmHg in which case the arm with higher BP was used. During the clinic visit, blood samples were taken for analysis, including lipid profiles. Diabetes was defined from the answer to the health questionnaire.

### Image acquisition

Participants underwent MRI scanning that included the acquisition of T1-weighted, T2-FLAIR and Time of Flight (ToF) angiography on a Philips 3T scanner according to the following acquisition protocol:

- T1-weighted—inversion-prepared gradient echo: repetition time 6.9 ms; echo time 3.1 ms; voxel size 1.09 × 1.09 × 1.0 mm^3^.
- FLAIR—repetition time 4,800 ms; inversion time 1,650 ms; echo time 125 ms; voxel size 1.09 × 1.09 × 1.0 mm^3^.
- Time of Flight angiography (ToF MRA)—repetition time 20 ms—echo time 3.5 ms—flip angle 20 degrees—voxel size 0.357 × 0.357 × 0.5 mm^3^.

### Image analysis

#### Visual assessment and manual annotation

All ToF MRA scans were visually assessed using multi-planar reconstruction, displaying cross-sections on axial, coronal and sagittal planes. The visual assessment was also crosschecked using 3D maximum intensity projection reconstructions.

Participants were first classified with respect to fetal variation of the circle of Willis (CoW). Three main subtypes were identified, following the classification of the posterior cerebral artery type described by Van Raamt et al. (19): complete CoW (group 1), transitional CoW (group 2) and fetal CoW (group 3). Then, four anatomical landmarks were identified at specified vascular branch-points and segments of the basilar artery were defined following established guidelines (20–22). As illustrated in Supplementary Figure 1, landmarks were annotated in a caudal direction in correspondence to: (1) the apex of the basilar artery; (2) the origin of the anterior inferior cerebellar artery (AICA) in the basilar artery; (3) the fusion point of the vertebral arteries; and (4) the origin of the posterior inferior cerebellar artery (PICA) in the larger vertebral artery.

If the AICA branched off the basilar artery asymmetrically, the origin of the larger AICA was labeled. Also, if an ipsilateral duplication of the AICA was observed, the more dominant AICA was labeled. If no PICA was found, either missing or outside the angiographic field of view, the landmark (4) was annotated at the most caudal position in the larger vertebral artery.

#### Basilar artery analysis and geometric markers

From MR angiography, the centerline of the basilar artery was delineated to connect the annotated anatomical landmarks following published numerical approaches (20, 21, 23). Then, the lumen was extracted along the vascular centerline as a sequence of closed contours of approximately circular shape (22). The actual length (the curved length) was computed considering the vascular centerline, whereas the median diameter was determined over the sequence of luminal contours. Given any two anatomical landmarks as defined above, the actual length was defined as the integral length of the connecting vascular centreline (physical length of the vascular branch). A representative diameter index was estimated by approximating and fitting the luminal closed contours with circular shapes. Given any two anatomical landmarks, a median diameter was determined for the underlying vascular branch, by considering the respective sequence of luminal closed contours and their associated diameters. To limit the number of variables that would be strongly correlated, the analysis in this study used the geometrical measurements of the whole annotated vascular branch, i.e., from the apex of the basilar artery to the origin of the PICA.

#### WMH analysis

White matter hyperintensities (WMH) were automatically segmented using a published framework (24). In short, an adaptive Gaussian mixture model was fitted to the rigidly registered T1 and FLAIR images. It allows for the modeling and presence of outliers and dynamically adapts the number of required components. At convergence, candidate lesion voxels are selected and the resulting connected components are automatically classified as lesion or artifacts based on their anatomical location and overall appearance. The aggregation of white matter and deep gray matter is further divided into subregions based on the closest cortical lobe and the normalized distance between ventricular and cortical surface divided in four equidistant layers.

**Figure 1:**
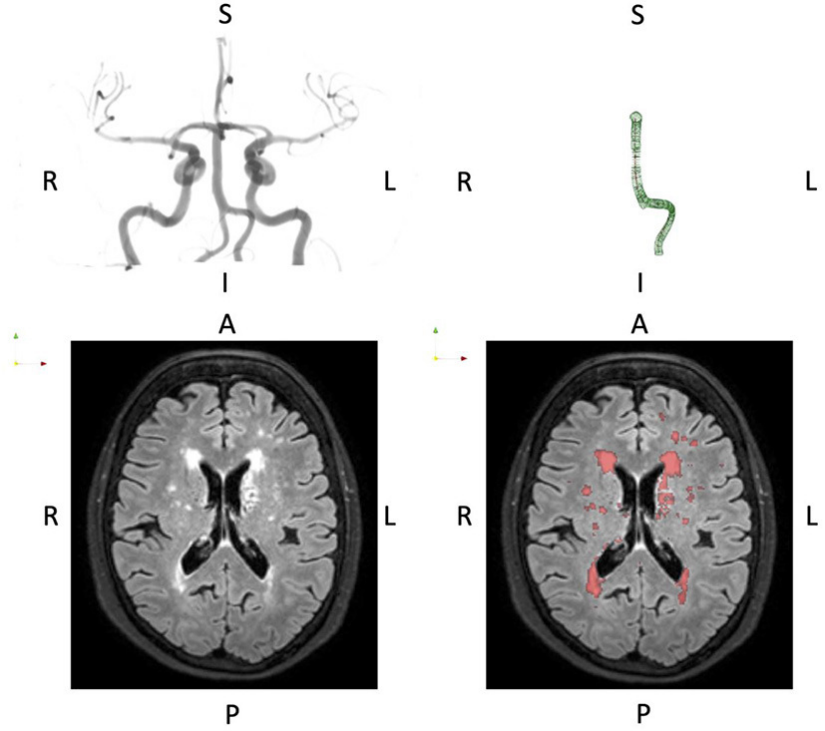
Typical presentation of basilar artery geometry (top row) and WMH burden (bottom row). The basilar artery segmentation is the full P1-P4 segment thus including the dominant vertebral artery segment. The orientation is indicated by the letters A, Anterior; P, Posterior; L, Left; R, Right; I, Inferior; S, Superior.

Figure 1 shows raw imaging data and associated processing for a typical subject.

### Statistical analysis

Data were compared across ethnic groups using the chi-square test for categorical data, ANOVA for continuous confounding variables and ANCOVA correcting a priori for age, sex and scale (total intracranial volume or height) for imaging derived data. Pairwise group differences were further assessed when differences were observed across all groups. WMH lesion load was log-transformed due to the skewness of the distribution. Multiple imputation (200 samples) was used to impute missing data. A summary of missingness is presented in Supplementary Table S1.

Linear regression models were constructed separately for each ethnic group to assess associations between BA metrics and predictors of interest. Separate models for each of the geometric markers of BA were fitted with these as dependent variables and demographic and cardiovascular risk factors of interest being the independent variables. For these analyses, cardiovascular risk factors included all variables used for the calculation of the Framingham risk score: systolic blood pressure, HDL and total cholesterol, age, sex, smoking status (ever- vs. never-smokers), and diabetes (binary variable). To this, we added diastolic blood pressure given the importance of BP in the etiology of CSVD and evidence for a greater contribution of diastolic BP to stroke risk in South Asian men(5). We added 10/5 mmHg to the systolic and diastolic measures, respectively, if the participant was on antihypertensive medication (25). In addition, we added lipid lowering (statin) medication, anti-hypertensive medication history of stroke (binary), history of cardiovascular disease (CHD) (binary), height, total intracranial volume (TIV), circle of Willis variant of posterior circulation (CoW 1 normal, CoW 2 transitional, CoW 3 fetal), and years of education to the models. Cohen's D effect size of each covariate in the linear regression was then reported.

These models were refitted replacing the dependent variable with log-transformed WMH volumes, replacing height by total intracranial volume and without considering the circle of Willis subtype.

Partial Spearman correlations were evaluated between geometric BA markers and WMH volumes adjusted first for age sex and TIV and then further including all confounders in each ethnic group to assess the independent association between large vessel characteristics and cerebral small vessel disease markers. Results are presented without correction for multiple comparisons.

## Results

### Population demographics, clinical and imaging variables across ethnicities

By original study design, there were more women in the African Caribbean group (Table 1) and African Caribbeans were slightly more often excluded due to basilar artery anatomy considerations (*p* = 0.039). South Asians and African Caribbeans were younger and shorter than Europeans, and were less likely to be ever-smokers. South Asians and African Caribbeans also had a higher prevalence of diabetes, and higher BP than Europeans. South Asians were more likely to report pre-existing coronary heart disease (CHD) than the other ethnic groups. While there was no difference across groups in terms of overall WMH volume (*p* = 0.192) and frontal load (*p* = 0.644), African Caribbeans displayed a larger parieto-occipital lesion load than the South Asian and European groups when correcting for age, sex and total intracranial volume (*p* = 0.015). When correcting for age, sex, height and circle of Willis subtype, African Caribbeans displayed a longer BA length than both South Asians and Europeans (*p* = 0.009).

**Table 1:** Demographic, clinical and imaging data stratified by ethnicity.

		**EU**	**SA**	**AC**	**P ANOVA**
Demographic	Female	41 (34.2)	53 (44.2)	79 (65.8)	<0.0005^b^^,^^c^
	Age (years)	73.6 (5.9)	72.9 (6.0)	70.5 (8.5)	0.001^b^
	Years education	12.3 (3.9)	12.6 (3.4)	11.7 (3.1)	0.204
	Height (cm)	169.5 (9.3)	162.3 (9.0)	163.8 (7.5)	<0.0005^a^^,^^b^
	Weight (kg)	79.4 (13.6)	70.5 (11.2)	80.8 (14.9)	<0.0005^a^^,^^c^
Clinical risk factors	Ever-smoker	56 (46.7)	3 (2.5)	21 (17.5)	<0.0005^a^^,^^b^^,^^c^
	Diabetes	16 (13.3)	35 (29.2)	29 (24.2)	0.011^a^^,^^b^
	Stroke	1 (0.8)	1 (0.8)	3 (2.5)	0.447
	Coronary Heart Disease	8 (6.7)	14 (11.7)	1 (0.9)	0.004^b^^,^^c^
	Statin use	52 (43.3)	79 (65.8)	40 (33.6)	<0.0005^a^^,^^c^
	Antihypertensive medication	48 (40.0)	86 (71.7)	77 (68.1)	<0.0005^a^^,^^b^
	Total cholesterol (mmol/l)	4.86 (1.07)	4.46 (1.07)	4.89 (1.08)	0.002^a^^,^^c^
	HDL-cholesterol (mmol/l)	1.58 (0.49)	1.51 (0.4)	1.8 (0.55)	<0.0005^b^^,^^c^
	Systolic BP (mmHg)	143 (18)	152 (17)	149 (17.8)	0.0001^a^^,^^b^
	Diastolic BP (mmHg)	81 (9.6)	84 (9.4)	85 (10.6)	0.023^a^^,^^b^
Imaging variables	CoW Variant	85 | 25 | 10	81 | 33 | 6	80 | 25 | 15	0.227
	TIV (mL)	1,382.63 (116.01)	1,241.65 (109.49)	1,236.87 (128.23)	<0.0005^a^^,^^b^
	BA Diameter (mm)^*^	2.90 [2.72; 3.10]	2.78 [2.60; 2.96]	2.82 [2.58; 2.96]	0.147
	BA Length (mm)^*^	48.16 [43.94; 54.48]	47.36 [43; 51.81]	51.29 [44.8; 56.49]	0.009^b^^,^^c^
	LesTot (mL)^*^	3.16 [1.66; 7.31]	2.50 [1.31; 7.18]	2.80 [1.56; 6.73]	0.193
	LesF (mL)^*^	1.68 [0.82; 3.88]	1.33 [0.67; 3.92]	1.59 [0.68; 3.81]	0.644
	LesPO (mL)^*^	0.75 [0.47; 2.04]	0.55 [0.28; 1.76]	0.89 [0.41; 2.06]	0.015^c^

Variables are reported as number (%) for categorical variables, mean (sd) for normally distributed variables and median (interquartile range) for skewed distributions. BA, basilar artery; BP, blood pressure; HDL, high density lipoprotein cholesterol; CoW Circle of Willis variants (i.e., complete, transitional and fetal subtypes); LesTot, LesF and LesPO indicate the total, frontal and parieto-occipital volumes of WMH, respectively; TIV, total intracranial volume. Significant differences comparing subgroups are noted

a (European vs. South Asian),

b (European vs African Caribbeans) and

c (South Asian vs African Caribbean). The

* indicates that data were a priori corrected for age, sex and scale to assess group differences.

### Association between demographic and cardiovascular risk factors and markers of EVA in the basilar artery

**Figure 2:**
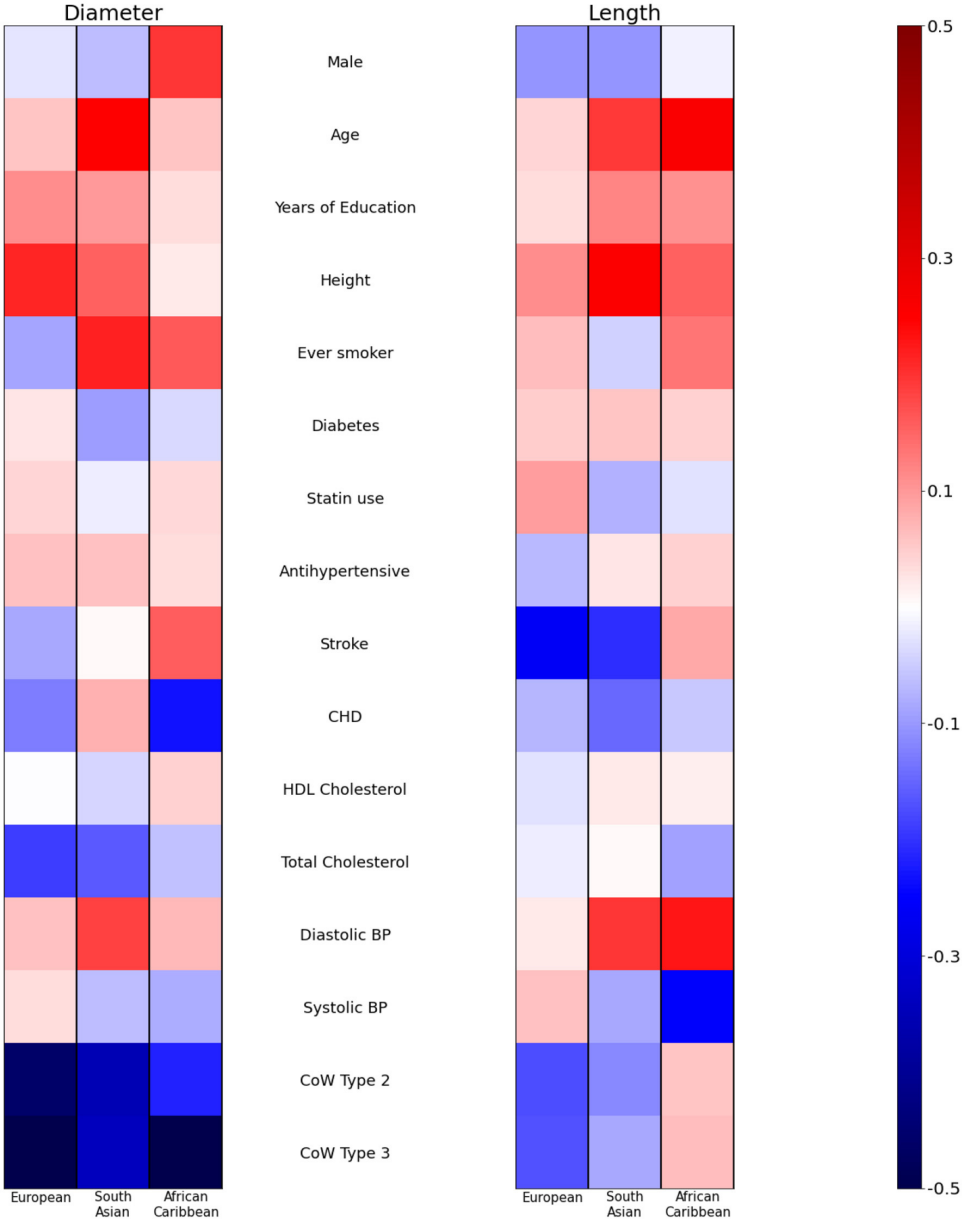
Cohen's D (effect size) of demographic and cardiovascular risk factors on basilar artery geometric characteristics. A separate model is fitted for each ethnicity adjusting simultaneously for all covariates. CoW, Circle of Willis; CHD, Coronary Heart Disease; BP, Blood pressure.

Figure 2 presents the Cohen's D effect size related to each of the co-adjusted demographic and cardiovascular risk factors included in the ethnic specific linear regression models. Coefficients of the model are available in Supplementary Table S4.

Older age was significantly associated with a greater BA diameter in South Asians (+11.3 μm/year [3.05; 19.62] *p* = 0.008) but not in other ethnic groups (+3.4 μm/year [−5.26; 12.12] (*p* = 0.436) in African Caribbeans and +2.6 μm/year [−5.75;10.87] (*p* = 0.543) in Europeans). Height was associated with a larger diameter in Europeans +7.74μm /cm [1.18; 14.31] (*p* = 0.021) and there was weak evidence of a similar association in South Asians +6.43 μm/cm [−1.11; 13.97] (*p* = 0.094), but this association was not evident in African Caribbeans −0.01μm/cm [−8.24; 8.22] (*p* = 0.998). Total cholesterol was negatively associated with diameter in Europeans −54.21 μm/mmol/L [−106.25; −2.17] (*p* = 0.041) with weak evidence of a similar negative association in South Asians −47.98 μm/mmol/L [−101.39; 5.43] (*p* = 0.078) but little evidence of an association in African Caribbeans −20.32 μm/mmol/L [−79.20; 38.57] (*p* = 0.494). There was evidence of a positive association between diastolic BP and diameter in South Asians (+7.626 μm/mmHg [0.13; 15.11] (*p* = 0.046) while there was no convincing evidence of an association in Europeans (2.79 μm/mmHg [−5.57; 11.15] (*p* = 0.510) or African Caribbeans (3.35 μm/mmHg [−5.29; 11.98] (*p* = 0.444).

Positive associations between age and length was observed in African Caribbeans +0.39 mm/year [0.12; 0.65] (*p* = 0.005) and South Asians +0.34 mm/year [0.02;0.65] (*p* = 0.037) but there was no convincing evidence of an association in Europeans (0.08 mm/year [−0.26; 0.41] (*p* = 0.653). Height was associated with BA length in South Asians (+0.33 mm/cm [0.09; 0.56] *p* = 0.006) but associations were unconvincing in Europeans (0.16 mm/cm [−0.10; 0.43] (*p* = 0.218) and African Caribbeans (0.21 mm/cm [−0.03; 0.44] (*p* = 0.091). Diastolic BP was associated with BA length in South Asians (+0.24 mm/mmHg [0.02; 0.45] (*p* = 0.034) and African Caribbeans 0.26 mm/mmHg [0.06; 0.47] (*p* = 0.013) with little evidence of an association in Europeans 0.03 mm/mmHg [−0.27; 0.34] (*p* = 0.827).

As expected from the neurovascular geometry, variants 2 and 3 of the circle of Willis were systematically associated with shorter and smaller basilar arteries, but the frequency of the variants did not differ by ethnicity (Table 1).

### Associations between demographic and cardiovascular risk factors and WMH volumes

**Figure 3:**
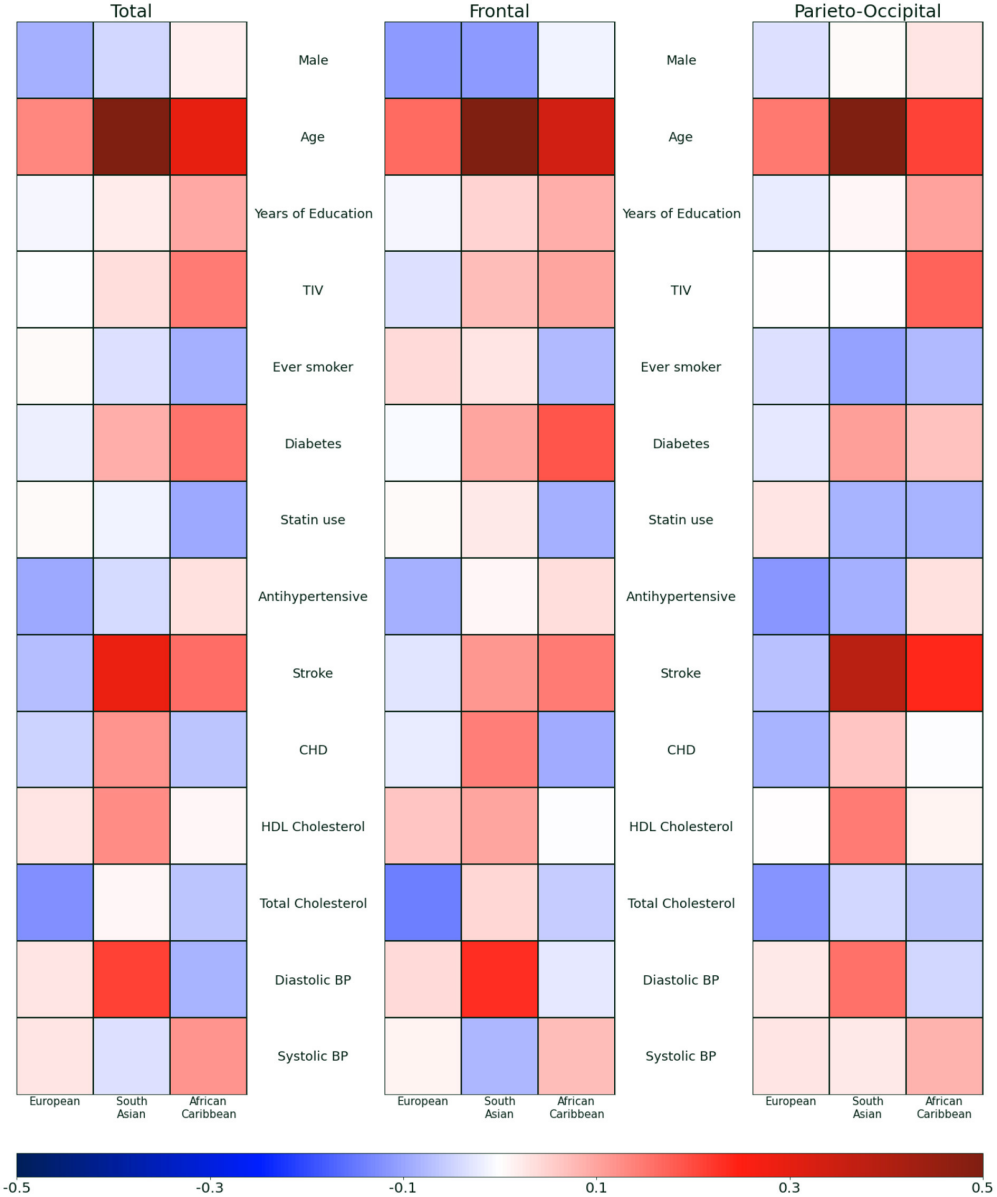
Effect size of demographic and cardiovascular risk factors on white matter hyperintensity volumes overall and in frontal and the parieto-occipital lobes. A separate model is fitted for each ethnicity adjusting simultaneously for all covariates. TIV, Total Intracranial Volume; BP, Blood pressure; CHD, Coronary Heart Disease.

Figure 3 presents the effect size related to each co-adjusted demographic and cardiovascular covariate in each ethnic group when investigating the association with WMH overall and in frontal and the parieto-occipital lobes separately. The quantitative values in percentage increase are presented in Supplementary Table S5.

Age was more strongly associated with WMH total and regional volumes in South Asians and African Caribbeans than Europeans (+8.40% [5.74; 11.13] per year of age (*p* < 0.0005) in South Asians, +4.41% [1.73; 7.15] (*p* = 0.001) in African Caribbeans compared with +2.61% [−0.85;6.18] (*p* = 0.140) in Europeans for total volume) (Supplementary Material). Diastolic BP was positively associated with total and regional WMH volumes in South Asians (+3.11% [0.45; 5.83] (*p* = 0.022) but there was no evidence of association in African Caribbeans −1.25% [−3.93; 1.50] per mmHg (*p* = 0.365) and in Europeans +0.52% [−2.68; 3.83] per mmHg (*p* = 0.750) for total volume). There was no evidence of association between systolic blood pressure and total WMH volume in either ethnic group (Europeans +0.31 [−1.54; 2.19] *p* = 0.742/South Asians −0.22 [−1.44; 1.01] *p* = 0.719/African Caribbean +1.07% [−0.54; 2.70] *p* = 0.191) despite a significant positive raw association between systolic blood pressure and total WMH volume prior to correction for other covariates (spearman Rho 25.26% *p* = 0.005) in the South Asian group.

### Association between BA characteristics and WMH lesion load by ethnicity

Table 2 presents the Spearman correlation coefficients when adjusting for age, sex and TIV for the WMH and age, sex, Height and CoW subtype for the BA (Model 1) and when further adjusting for all potential confounders (Model 2).

**Table 2:** Partial Spearman correlation coefficients presented as % (*p*-value) between basilar artery characteristics and WMH volumes after correction for age, sex, CoW variants and height/TIV for Model 1 and further adjusting for demographic and cardiovascular confounders (Model 2).

	**Diameter**			**Length**		
	**Total**	**Frontal**	**Parieto-occipital**	**Total**	**Frontal**	**Parieto-occipital**
**Model 1**						
EU	15.29 (0.095)	16.63 (0.070)	8.18 (0.374)	−12.28 (0.181)	−10.79 (0.241)	−11.84 (0.198)
SA	17.89 (0.051)	20.95 (0.022)	6.31 (0.493)	24.81 (0.006)	26.18 (0.004)	26.08 (<0.0001)
AC	9.70 (0.292)	12.32 (0.180)	10.56 (0.251)	−13.33 (0.147)	−11.78 (0.200)	−11.31 (0.219)
**Model 2**						
EU	14.55 (0.113)	15.85 (0.084)	6.11 (0.507)	−16.72 (0.068)	−15.81 (0.085)	−17.65 (0.054)
SA	17.64 (0.054)	21.31 (0.019)	5.41 (0.557)	22.62 (0.013)	23.16 (0.011)	21.71 (0.017)
AC	11.73 (0.202)	12.63 (0.169)	10.42 (0.258)	−17.09 (0.062)	−17.20 (0.060)	−15.79 (0.085)

EU, European; SA, South Asian; AC, African Caribbean; TIV, Total intracranial volume; WMH, White matter hyperintensity; CoW, Circle of Willis variant.

A positive association between diameter of the basilar artery and white matter hyperintensity volume was seen in all ethnic groups, however this only reached significance for the South Asian sample (rho = 17.89 *p* = 0.05) compared to Europeans (15.29 *p* = 0.10) and African Caribbeans (rho = 9.70 *p* = 0.29). In turn, the relationship between length and WMH volume varied greatly across ethnicities. While length was positively associated with WMH volume in South Asians (rho = 24.81%, *p* = 0.010), evidence for a similar association was almost inverted in Europeans (rho = −12.28, *p* = 0.18) or African Caribbeans (rho = −13.33, *p* = 0.15). All associations were essentially unaltered by multivariable adjustment for potential confounders.

## Discussion

We found ethnic differences in EVA, WMH and inter-associations between EVA in the basilar artery and WMH. These differences were not explained by cardiovascular risk factors and or other demographic confounders. It is noteworthy, that the observed associations with WMH were not limited to the territories supplied by the basilar artery. This is consistent with the basilar artery measures being representative of more global cerebral EVA.

We found a number of ethnic group differences in cerebral EVA across ethnicities when adjusting for age, sex and scale and in the individual patterns of associations. This was the case for both the influence of cardiovascular risk factors on basilar artery measures, as well as for the interrelationships between basilar artery measures and small vessel disease. Specifically, we found an equivalent increase of 1 year in age was associated with a 3- to 4-fold greater dilation of the basilar artery in South Asians than in Europeans and African Caribbeans, respectively. Similarly, age and increased diastolic BP each had the most adverse effect on WMH volume in South Asians. This replicates findings on another subsample of the SABRE study highlighting differences in the association of local WMH burden with age and diastolic blood pressure in South Asians when compared to Europeans (26). This relationhip with diastolic blood pressure must however be put in perpective as the proportion of individuals with hypertension is particularly high in the South Asian subgroup. This may explain the observed direction of relationship in apparent disagreement with studies with lower proportion of hypertensive subjects (27). Interestingly, the relationship between scale (height) and diameter only remained significant in the European group after correction for other confounders. This may reflect a possible sex-dependent relationship as this was also the group with the highest proportion of male. In comparison, a relationship between diameter and male sex was only significant in the African-Caribbean group where the proportion of male participants was the lowest. These findings may warrant further sex-specific analysis in the study of EVA.

Finally, while we found a positive association between diameter and WMH volumes across all ethnic groups (reaching significance for the South Asian group), a positive association with length was only found in the South Asians. This echoes findings from Rundek et al. (28) linking carotid artery diameter and white matter hyperintensities in another multi-ethnic study with a large proportion of individuals of Hispanic descent. We also found that higher diastolic BP had a strong negative impact in South Asian people.

Taking these findings together, our work suggests that the basilar artery is particularly susceptible to the adverse effects of aging in South Asians, while there is evidence of a more marked association between age and length in African Caribbeans. These observations are consistent with the stronger associations between age and CSVD in South Asians and African Caribbeans, and their greater susceptibility to stroke (5) and, in the case of South Asians, white matter disease (26). Differences in remodeling patterns between South Asians and African Caribbeans might indicate that EVA is more severe in South Asians, since axial changes appear to be among the earlier manifestations of EVA (14). Alternatively it could reflect differences in underlying mechanisms with African Caribbeans being less susceptible to atherosclerosis than South Asians (1, 29, 30) with higher BP playing a more important role in people of Black African descent (31). In contrast atherosclerosis of large intracranial arteries may make a larger contribution to strokes in people of South Asian origin (32). Dilatation and length of the basilar artery were observed as risk factors for CSVD and CVD in previous studies in a Japanese population (16) but we did not observe a similar correlation with CSVD in African Caribbeans, whether this is linked to the strong influence of BP and the low prevalence of atherosclerosis in this ethnic group remains speculative.

Our study has limitations. Causal relationships could not be inferred given the cross-sectional design of the study. Although only the WMH burden was considered in the evaluation of cerebral small vessel disease, a number of additional indicators (e.g., number of lacunes, cerebral microbleeds and extent of enlarged perivascular spaces) should be included in future analyses to provide a more complete picture of pathology given notably that lacunar infarcts have been reported to be more prevalent in South Asian populations. Measures of arterial stiffness through pulse wave velocity could be also analyzed in relationship with basilar artery characteristics. Further, beyond ethnic differences, cardiovascular risk factors and demographics characteristics considered in this study, environmental factors (33) and epigenetic changes themselves related to vascular aging (34) could influence our findings. Strengths of the study include the population-based tri-ethnic sample from a community with publicly-funded universal access to healthcare, and use of systematic deep phenotyping to characterize both vasculature and brain damage.

This study provides evidence of differential EVA by ethnicity, and we suggest this may provide an explanation for the unexplained elevated stroke and dementia risk in South Asians and African Caribbean people. The greater burden of WMH in South Asians highlights the need for population specific normative data when assessing cerebrovascular risk factors and studying the pathophysiological pathways of cerebral small vessel disease and age-related vascular changes.

## Data availability statement

The datasets presented in this article are not readily available because the data that support the findings of this study are available from the SABRE study (https://www.sabrestudy.org/) upon reasonable request. Requests to access the datasets should be directed to sabre@ucl.ac.uk.

## Ethics statement

The studies involving human participants were reviewed and approved by National Research Ethics Service Committee, London-Fulham (14/LO/0108). The patients/participants provided their written informed consent to participate in this study.

## Author contributions

Study concept and design: CS, AH, NC, HJ, and MC. Acquisition of the data: LS, DA, HJ, and TT. Analysis and interpretation of the data: CS, SM, RR, HJ, MC, NC, AH, and TT. Drafting of the manuscript: CS. All authors revised the manuscript for important intellectual content. All authors contributed to the article and approved the submitted version.

## Funding

CS was funded by Alzheimer's Society (AS-JF-17-011). SM, MC, and SO received funding from Wellcome/EPSRC Centre for Medical Engineering (WT203148/Z/16/Z) and Wellcome Flagship Programme (WT213038/Z/18/Z). The Dementia Research Centre was supported by Alzheimer's Research UK, Brain Research Trust, and The Wolfson Foundation. MC received funding from EPSRC (EP/H046410/1). The SABRE study was funded at baseline by the UK Medical Research Council, Diabetes UK, the British Heart Foundation, and at follow-up by the Wellcome Trust (WT082464), British Heart Foundation (SP/07/001/23603 and CS/13/1/30327), and Diabetes UK (13/0004774). JB was supported by Alzheimer's Research UK - ARUK-SRF2016A-2.

## Conflict of interest

The authors declare that the research was conducted in the absence of any commercial or financial relationships that could be construed as a potential conflict of interest. The reviewer JC declared a shared affiliation with some of the authors, CS, SM, SO, and MC, to the handling editor at time of review.

## Publisher's note

All claims expressed in this article are solely those of the authors and do not necessarily represent those of their affiliated organizations, or those of the publisher, the editors and the reviewers. Any product that may be evaluated in this article, or claim that may be made by its manufacturer, is not guaranteed or endorsed by the publisher.

## Abbreviations

AC: African-Caribbean
BA: Basilar artery
BP: Blood pressure
CSVD: Cerebral small vessel disease
CoW: Circle of Willis
EU: European
SA: South-Asian
TIV: Total intracranial volume
WMH: White matter hyperintensities.
