# Supplementary material for: Accelerated vascular aging: Ethnic differences in basilar artery length and diameter, and its association with cardiovascular risk factors and cerebral small vessel disease
Source: Front Cardiovasc Med. 2022 Jul 28;9:939680. doi: 10.3389/fcvm.2022.939680 (PMC9366336; doi:10.3389/fcvm.2022.939680)
Supplement: Supplementary file 1 [file Data_Sheet_1.docx]

*Accelerated Vascular ageing:* ethnic differences in basilar artery geometry, and its relations to cardiovascular risk factors and cerebral small vessel disease

Carole H Sudre^1,2,3^, Stefano Moriconi^3^, Rafael Rehwald^4^, Lorna Smith^5^, Therese Tillin^1^, Josephine Barnes^6^, David Atkinson^5^, Sébastien Ourselin^3^, Nish Chaturvedi^1^, Alun Hughes^1^, H Rolf Jäger^7*^, M Jorge Cardoso^3*^

# Supplementary material –

Figure S1 - Example of location of the 4 landmarks defining the different segments of the basilar artery P1: the apex of the basilar artery; P2 origin of the anterior inferior cerebellar artery (AICA) in the basilar artery; P3: fusion point of the vertebral arteries – note that the second vertebral artery is hidden in this view; P4 origin of the posterior inferior cerebellar artery (PICA) in the larger vertebral artery. Orientation of the image is indicated by the letters L – Left ; R – Right; I – Inferior; S - Superior


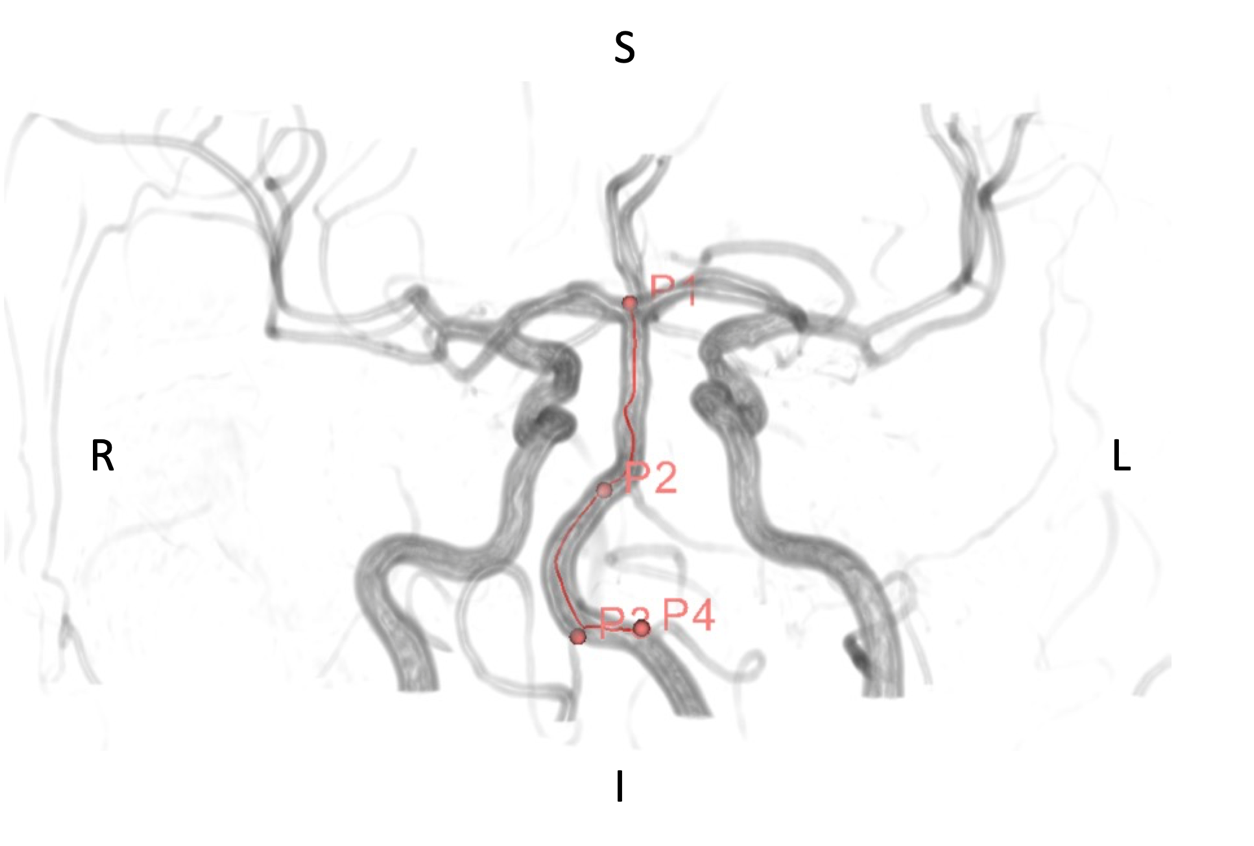


Table S1 - Record of missing data across ethnicities. AC – African Caribbean; EU – European; SA – South Asian

|  | **EU** | **SA** | **AC** |
| --- | --- | --- | --- |
| **Stroke** | 1 | 2 | 1 |
| **Coronary Heart disease** | 0 | 0 | 5 |
| **Antihypertensive use** | 0 | 0 | 7 |
| **Statin use** | 0 | 0 | 1 |
| **HDL - cholesterol** | 2 | 0 | 1 |
| **Total Cholesterol** | 2 | 0 | 1 |
| **Years of education** | 21 | 40 | 36 |

Table S2 - Univariate Spearman correlation coefficients (p values) for the relationship between variables and basilar artery characteristics. Acronyms: AC – African Caribbean; EU – European; SA – South Asian; TIV – Total Intracranial Volume; CHVD – Coronary Heart Disesase; BP – Blood pressure;

|  | Diameter | | | Length | | |
| --- | --- | --- | --- | --- | --- | --- |
|  | EU | SA | AC | EU | SA | AC |
| Male | 23.76 (0.009) | 28.70 (0.001) | 26.05 (0.004) | 5.81 (0.529) | 11.99 (0.192) | 23.21 (0.011) |
| Age | 4.41 (0.632) | 23.54 (0.010) | 12.54 (0.172) | 2.23 (0.809) | 8.04 (0.383) | 18.70 (0.041) |
| Years of education | 0.48 (0.962) | 14.61 (0.196) | 5.14 (0.643) | 8.88 (0.382) | 12.93 (0.253) | 5.52 (0.618) |
| TIV | 21.20 (0.02) | 18.78 (0.040) | 19.97 (0.029) | 8.67 (0.346) | 8.76 (0.341) | 5.80 (0.529) |
| Height | 25.8 (0.004) | 24.60 (0.007) | 13.21 (0.150) | 12.86 (0.161) | 22.74 (0.012) | 19.40 (0.034) |
| Smoking | 4.63 (0.616) | 15.33 (0.095) | 15.99 (0.081) | 19.96 (0.029) | -1.16 (0.900) | 17.63 (0.054) |
| Diabetes | 0.21 (0.982) | -3.68 (0.690) | 0.59 (0.949) | -1.56 (0.866) | 4.63 (0.615) | 5.14 (0.577) |
| Statin | 12.57 (0.171) | 6.01 (0.514) | 15.12 (0.101) | 13.35 (0.146) | -8.39 (0.362) | -0.05 (0.996) |
| Antihypertensive | 20.38 (0.026) | 17.62 (0.054) | 12.27 (0.285) | 2.31 (0.802) | -2.56 (0.781) | 5.45 (0.636) |
| Stroke | 3.48 (0.707) | 11.54 (0.214) | 3.12 (0.736) | -6.97 (0.451) | 6.38 (0.493) | 2.65 (0.775) |
| CHD | 4.73 (0.608) | 16.94 (0.064) | -14.39 (0.125) | 4.53 (0.623) | -10.87 (0.237) | 8.46 (0.368) |
| HDL cholesterol | -10.56 (0.255) | -8.92 (0.333) | -12.41 (0.289) | -5.79 (0.534) | 0.52 (0.955) | 2.10 (0.858) |
| Total cholesterol | -25.83 (0.005) | -24.45 (0.007) | -12.63 (0.280) | -11.84 (0.202) | 0.33 (0.972) | -23.42 (0.043) |
| Diastolic BP | 11.82 (0.198) | 4.92 (0.594) | -7.68 (0.405) | 2.37 (0.797) | 14.63 (0.111) | 8.30 (0.368) |
| Systolic BP | 9.44 (0.305) | 11.98 (0.192) | -2.07 (0.822) | -0.77 (0.933) | 8.71 (0.344) | -3.27 (0.723) |
| CoW variant | -48.98 (<0.001) | -38.57 (<0.0001) | -43.37 (<0.0001) | -20.04 (0.028) | -14.66 (0.110) | 5.78 (0.530) |

Table S3 - Univariate Spearman correlation coefficients (p values) between lesion load and demographic and cardiovascular risk factors. Acronyms: AC – African Caribbean; EU – European; SA – South Asian; TIV – Total Intracranial Volume; CHVD – Coronary Heart Disesase; BP – Blood pressure;

|  | Total | | | Frontal | | | Parieto-Occipital | | |
| --- | --- | --- | --- | --- | --- | --- | --- | --- | --- |
|  | EU | SA | AC | EU | SA | AC | EU | SA | AC |
| Male | -2.97 (0.748) | 8.04 (0.383) | 7.03 (0.446) | -8.60 (0.350) | 4.65 (0.614) | 6.67 (0.469) | 5.35 (0.562) | 8.33 (0.366) | 9.41 (0.307) |
| Age | 14.15 (0.123) | 39.41 (<0.0001) | 36.42 (<0.0001) | 15.38 (0.093) | 40.41 (<0.0001) | 41.36 (<0.0001) | 15.01 (0.102) | 36.88 (<0.0001) | 24.89 (0.006) |
| Years of education | 3.11 (0.760) | 7.15 (0.528) | 16.71 (0.129) | 3.04 (0.766) | 9.58 (0.398) | 12.27 (0.266) | -2.47 (0.809) | 3.64 (0.749) | 18.06 (0.100) |
| TIV | -1.00 (0.914) | 8.85 (0.337) | 9.54 (0.300) | -6.49 (0.482) | 9.33 (0.311) | 4.99 (0.588) | 2.73 (0.767) | 4.88 (0.597) | 15.79 (0.085) |
| Height | -10.7 (0.245) | -0.62 (0.946) | -10.32 (0.262) | -13.59 (0.139) | -5.25 (0.569) | -12.86 (0.162) | -6.97 (0.449) | 4.01 (0.664) | -5.77 (0.531) |
| Smoking | -0.68 (0.942) | 1.93 (0.835) | -10.29 (0.263) | 0.87 (0.925) | 2.54 (0.783) | -9.02 (0.327) | -0.87 (0.925) | -6.70 (0.467) | -10.73 (0.243) |
| Diabetes | 3.40 (0.713) | 6.62 (0.473) | 12.84 (0.162) | 5.80 (0.529) | 7.41 (0.421) | 16.77 (0.067) | 0.07 (0.994) | 8.26 (0.37) | 7.61 (0.408) |
| Statin | 6.99 (0.448) | 3.63 (0.694) | 1.66 (0.858) | 8.40 (0.362) | 7.43 (0.420) | 3.78 (0.683) | 9.56 (0.299) | -3.32 (0.719) | -1.04 (0.911) |
| Antihypertensive | -10.21 (0.267) | 9.96 (0.279) | 18.85 (0.098) | -10.02 (0.276) | 14.01 (0.127) | 20.77 (0.068) | -12.57 (0.171) | 5.53 (0.549) | 17.89 (0.117) |
| Stroke | -2.68 (0.772) | 15.61 (0.091) | 8.27 (0.371) | -1.61 (0.862) | 15.61 (0.091) | 7.18 (0.438) | -1.07 (0.908) | 15.88 (0.086) | 12.33 (0.182) |
| CHD | -4.92 (0.594) | 17.46 (0.056) | -1.69 (0.857) | -1.25 (0.892) | 18.29 (0.046) | -5.08 (0.59) | -9.35 (0.309) | 9.89 (0.282) | 1.97 (0.834) |
| HDL cholesterol | 5.71 (0.539) | 8.08 (0.380) | 5.54 (0.637) | 10.82 (0.244) | 6.70 (0.467) | 3.99 (0.734) | 3.75 (0.686) | 7.67 (0.405) | 5.45 (0.642) |
| Total cholesterol | -7.85 (0.398) | -0.25 (0.978) | -21.75 (0.061) | -9.59 (0.301) | -1.49 (0.872) | -24.65 (0.033) | -10.18 (0.273) | 0.98 (0.916) | -17.24 (0.139) |
| Diastolic BP | -1.03 (0.912) | 16.49 (0.072) | -2.18 (0.813) | -2.08 (0.822) | 15.48 (0.091) | -3.09 (0.737) | -0.94 (0.919) | 20.82 (0.022) | 3.22 (0.727) |
| Systolic BP | 1.74 (0.850) | 25.26 (0.005) | 16.52 (0.071) | 0.51 (0.956) | 21.84 (0.017) | 19.43 (0.033) | 3.76 (0.684) | 28.95 (0.001) | 14.17 (0.123) |
| CoW variant | 3.65 (0.692) | 6.98 (0.449) | -13.60 (0.139) | 2.30 (0.803) | 5.85 (0.526) | -14.13 (0.124) | 4.29 (0.641) | 10.30 (0.263) | -14.56 (0.113) |

Table S4 - Linear model relating BA characteristics and demographic and cardiovascular risk factors. Regression coefficients (Beta) are presented as *μ*m change in diameter or mm change in length per unit change of independent variable and Cohen’s D effect size (ES). Acronyms: BP = blood pressure; CI=Confidence interval; CoW Circle of Willis variant

|  |  | **European** | | | **South Asian** | | | **African Carribbean** | | |
| --- | --- | --- | --- | --- | --- | --- | --- | --- | --- | --- |
|  |  | **Beta 95% CI** | **ES** | **P-value** | **Beta 95% CI** | **ES** | **P-value** | **Beta 95% CI** | **ES** | **P-value** |
| Diameter | **Sex** | -20.34  [-169.60 ; 128.93] | -0.025 | 0.787 | -55.47  [-215.03 ; 104.09] | -0.063 | 0.492 | **148.19**  **[12.86 ; 283.51]** | **0.198** | **0.032** |
| Diameter | **Age** | 2.56  [-5.75 ; 10.87] | 0.056 | 0.543 | **11.34**  **[3.05 ; 19.62]** | **0.248** | **0.008** | 2.72  [-5.89 ; 11.34] | 0.057 | 0.532 |
|  | **Years Education** | 9.41  [-5.86 ; 24.69] | 0.112 | 0.224 | 9.03  [-7.32 ; 25.37] | 0.100 | 0.275 | 3.20  [-15.21 ; 21.61] | 0.032 | 0.730 |
|  | **TIV** | **7.74**  **[1.18 ; 14.31]** | **0.214** | **0.021** | 6.43  [-1.11 ; 13.97] | 0.155 | 0.094 | 1.02  [-7.07 ; 9.11] | 0.023 | 0.803 |
|  | **Smoking** | -46.34  [-141.88 ; 49.20] | -0.088 | 0.338 | **253.99**  **[42.59 ; 465.39]** | **0.218** | **0.019** | 119.36  [-15.22 ; 253.93] | 0.161 | 0.082 |
|  | **Diabetes** | 27.16  [-155.82 ; 210.14] | 0.027 | 0.769 | -54.97  [-157.07 ; 47.13] | -0.098 | 0.288 | -23.00  [-136.59 ; 90.59] | -0.037 | 0.689 |
|  | **Statin** | 27.99  [-100.31 ; 156.3] | 0.040 | 0.666 | -10.36  [-130.01 ; 109.29] | -0.016 | 0.864 | 21.57  [-85.13 ; 128.27] | 0.037 | 0.689 |
|  | **Antihypertensive** | 37.17  [-71.79 ; 146.12] | 0.062 | 0.5 | 43.09  [-83.38 ; 169.56] | 0.062 | 0.501 | 25.20  [-111.89 ; 162.29] | 0.034 | 0.715 |
|  | **Stroke** | -74.08  [-233.09 ; 84.93] | -0.085 | 0.356 | 6.17  [-237.03 ; 249.38] | 0.005 | 0.960 | 192.79  [-29.04 ; 414.63] | 0.157 | 0.088 |
|  | **CHD** | -179.22  [-432.79 ; 74.36] | -0.128 | 0.164 | 58.81  [-84.21 ; 201.82] | 0.074 | 0.417 | **-599.16**  **[-1078.37 ; -119.94]** | **-0.231** | **0.016** |
|  | **HDL-Cholesterol** | -1.68  [-102.52 ; 99.16] | -0.003 | 0.974 | -26.19  [-141.22 ; 88.84] | -0.041 | 0.652 | 23.49  [-73.48 ; 120.45] | 0.044 | 0.631 |
|  | **Cholesterol** | **-54.21**  **[-106.25 ; -2.17]** | **-0.189** | **0.041** | -47.98  [-101.39 ; 5.43] | -0.163 | 0.078 | -19.46  [-77.44 ; 38.53] | -0.061 | 0.506 |
|  | **Diastolic BP** | 2.79  [-5.57 ; 11.15] | 0.060 | 0.51 | **7.62**  **[0.13 ; 15.11]** | **0.184** | **0.046** | 3.19  [-5.41 ; 11.80] | 0.067 | 0.464 |
|  | **Systolic BP** | 0.79 [-3.39 ; 4.96] | 0.034 | 0.71 | -1.53  [-5.95 ; 2.89] | -0.063 | 0.493 | -2.07  [-6.76 ; 2.62] | -0.080 | 0.383 |
|  | **CoW2** | **-276.61**  **[-385.87 ; -167.35]** | **-0.458** | **<0.0001** | **-209.01**  **[-316.10 ; -101.91]** | **-0.353** | **<0.0001** | **-147.11**  **[-271.01 ; -23.20]** | **-0.215** | **0.020** |
|  | **CoW3** | **-599.79**  **[-813.90 ; -385.69]** | **-0.507** | **<0.0001** | **-467.46**  **[-716.14 ; -218.78]** | **-0.340** | **<0.0001** | **-440.77**  **[-579.70 ; -301.84]** | **-0.574** | **<0.0001** |
| Length | **Sex** | -3.11 [-8.62 ; 2.39] | -0.102 | 0.265 | -2.65 [-7.22 ; 1.93] | -0.105 | 0.254 | -0.34 [-4.90 ; 4.22] | -0.014 | 0.882 |
|  | **Age** | 0.08 [-0.26 ; 0.41] | 0.041 | 0.653 | **0.34 [0.02 ; 0.65]** | **0.193** | **0.037** | **0.39 [0.12 ; 0.66]** | **0.261** | **0.005** |
|  | **Years Education** | 0.11 [-0.48 ; 0.71] | 0.035 | 0.704 | 0.33 [-0.18 ; 0.85] | 0.118 | 0.199 | 0.30 [-0.20 ; 0.79] | 0.109 | 0.238 |
|  | **TIV** | 0.16 [-0.10 ; 0.43] | 0.113 | 0.218 | **0.33 [0.09 ; 0.56]** | **0.255** | **0.006** | 0.21 [-0.04 ; 0.45] | 0.153 | 0.097 |
|  | **Smoking** | 1.46 [-2.60 ; 5.53] | 0.065 | 0.477 | -1.82 [-8.96 ; 5.32] | -0.046 | 0.614 | 3.60 [-1.17 ; 8.37] | 0.137 | 0.138 |
|  | **Diabetes** | 2.01 [-5.45 ; 9.48] | 0.049 | 0.593 | 0.99 [-2.27 ; 4.26] | 0.055 | 0.548 | 0.89 [-2.83 ; 4.61] | 0.043 | 0.635 |
|  | **Statin** | 3.10 [-2.66 ; 8.86] | 0.098 | 0.288 | -1.70 [-5.82 ; 2.41] | -0.075 | 0.414 | -0.58 [-4.18 ; 3.02] | -0.029 | 0.749 |
|  | **Antihypertensive** | -1.56 [-5.65 ; 2.53] | -0.069 | 0.45 | 0.58 [-3.63 ; 4.80] | 0.025 | 0.785 | 1.03 [-3.04 ; 5.10] | 0.046 | 0.616 |
|  | **Stroke** | -7.35 [-12.46 ; -2.24] | -0.261 | 0.005 | **-8.11 [-15.28 ; -0.95]** | **-0.205** | **0.027** | 2.45 [-2.80 ; 7.70] | 0.085 | 0.356 |
|  | **Coronary heart disease** | -3.12 [-11.11 ; 4.88] | -0.071 | 0.441 | -3.98 [-8.96 ; 0.99] | -0.145 | 0.115 | -2.73 [-12.30 ; 6.84] | -0.052 | 0.571 |
|  | **HDLCholesterol** | -0.73 [-5.20 ; 3.75] | -0.029 | 0.748 | 0.52 [-3.75 ; 4.78] | 0.022 | 0.810 | 0.37 [-3.52 ; 4.27] | 0.017 | 0.849 |
|  | **Cholesterol** | -0.24 [-2.79 ; 2.30] | -0.017 | 0.851 | 0.06 [-1.96 ; 2.08] | 0.006 | 0.952 | -0.93 [-2.79 ; 0.93] | -0.090 | 0.325 |
|  | **Diastolic BP** | 0.03 [-0.27 ; 0.34] | 0.02 | 0.827 | **0.24 [0.02 ; 0.45]** | **0.196** | **0.034** | **0.26 [0.06 ; 0.47]** | **0.230** | **0.013** |
|  | **Systolic BP** | 0.05 [-0.10 ; 0.20] | 0.059 | 0.522 | -0.05 [-0.17 ; 0.06] | -0.083 | 0.366 | **-0.20 [-0.35 ; -0.06]** | **-0.250** | **0.007** |
|  | **CoW2** | -4.17 [-8.48 ; 0.14] | -0.175 | 0.058 | -2.12 [-5.41 ; 1.18] | -0.116 | 0.205 | 1.36 [-3.01 ; 5.74] | 0.056 | 0.538 |
|  | **CoW3** | -7.20 [-14.87 ; 0.48] | -0.170 | 0.066 | -2.96 [-9.29 ; 3.36] | -0.085 | 0.354 | 1.60 [-3.00 ; 6.20] | 0.063 | 0.491 |

Table S5 -Linear model relating WMH characteristics and demographic and cardiovascular risk factors. Regression coefficients (Beta) are presented as *%* change per unit change of independent variable and Cohen’s D effect size (ES). Acronyms: BP = blood

|  |  | **European** | | | **South Asian** | | | **African Carribean** | | |
| --- | --- | --- | --- | --- | --- | --- | --- | --- | --- | --- |
|  |  | **Beta 95% CI** | **ES** | **P-value** | **Beta 95% CI** | **ES** | **P-value** | **Beta 95% CI** | **ES** | **P-value** |
| **Total lesion** | **Sex** | -22.81 [-54.36 ; 30.56] | -0.089 | 0.331 | -10.85 [-43.15 ; 39.79] | -0.046 | 0.614 | 5.41 [-35.70 ; 72.81] | 0.019 | 0.833 |
|  | **Age** | 2.61 [-0.85 ; 6.18] | 0.136 | 0.140 | **8.40 [5.74 ; 11.13]** | **0.588** | **<0.0001** | **4.43 [1.70 ; 7.24]** | **0.297** | **0.002** |
|  | **Years Education** | -0.30 [-5.75 ; 5.47] | -0.010 | 0.915 | 0.68 [-4.65 ; 6.31] | 0.023 | 0.804 | 4.45 [-3.76 ; 13.35] | 0.097 | 0.293 |
|  | **TIV** | 0 [0 ; 0] | -0.001 | 0.991 | 0 [0 ; 0] | 0.038 | 0.679 | 0 [0 ; 0] | 0.148 | 0.107 |
|  | **Smoking** | 0.97 [-32.25 ; 50.48] | 0.004 | 0.962 | -9.77 [-47.28 ; 54.43] | -0.035 | 0.705 | -24.19 [-57.46 ; 35.09] | -0.087 | 0.344 |
|  | **Diabetes** | -5.56 [-44.90 ; 61.86] | -0.019 | 0.834 | 19.17 [-16.59 ; 70.27] | 0.089 | 0.332 | 47.44 [-6.45 ; 132.37] | 0.155 | 0.094 |
|  | **Statin** | 1.29 [-37.57 ; 64.36] | 0.005 | 0.958 | -3.57 [-36.80 ; 47.13] | -0.016 | 0.865 | -22.68 [-52.03 ; 24.61] | -0.098 | 0.287 |
|  | **Antihypertensive** | -23.60 [-54.20 ; 27.44] | -0.095 | 0.299 | -8.39 [-38.00 ; 35.38] | -0.041 | 0.657 | 11.02 [-36.29 ; 93.47] | 0.034 | 0.708 |
|  | **Stroke** | -19.43 [-53.13 ; 38.5] | -0.072 | 0.431 | 188.12 [47.47 ; 462.93] | 0.287 | 0.002 | 126.63 [-9.05 ; 464.72] | 0.162 | 0.078 |
|  | **CHD** | -19.68 [-65.21 ; 85.46] | -0.047 | 0.605 | 54.12 [-20.11 ; 197.33] | 0.119 | 0.195 | -34.65 [-80.11 ; 114.74] | -0.065 | 0.478 |
|  | **HDLCholesterol** | 7.38 [-30.09 ; 64.92] | 0.030 | 0.743 | 31.22 [-11.02 ; 93.53] | 0.127 | 0.168 | 2.80 [-34.72 ; 61.87] | 0.011 | 0.904 |
|  | **Cholesterol** | -14.95 [-32.96 ; 7.9] | -0.123 | 0.180 | 1.12 [-16.83 ; 22.95] | 0.010 | 0.91 | -9.48 [-31.11 ; 18.94] | -0.066 | 0.47 |
|  | **Diastolic** | 0.52 [-2.68 ; 3.83] | 0.029 | 0.750 | **3.11 [0.45 ; 5.83]** | **0.213** | **0.022** | -1.25 [-3.93 ; 1.5] | -0.083 | 0.366 |
|  | **Systolic** | 0.31 [-1.54 ; 2.19] | 0.030 | 0.742 | -0.22 [-1.44 ; 1.01] | -0.033 | 0.719 | 1.07 [-0.54 ; 2.7] | 0.120 | 0.192 |
| **Frontal** | **Sex** | -28.24 [-57.81 ; 22.07] | -0.113 | 0.218 | -26.06 [-55.13 ; 21.83] | -0.109 | 0.233 | -4.39 [-45.18 ; 66.74] | -0.015 | 0.873 |
|  | **Age** | 3.07 [-0.29 ; 6.54] | 0.165 | 0.074 | **8.89 [6.07 ; 11.79]** | **0.588** | **<0.0001** | **6.03 [2.76 ; 9.4]** | **0.339** | **<0.0001** |
|  | **Years Education** | -0.30 [-5.87 ; 5.61] | -0.009 | 0.919 | 1.51 [-4.1 ; 7.45] | 0.048 | 0.602 | 4.39 [-4.5 ; 14.11] | 0.088 | 0.339 |
|  | **TIV** | 0 [0 ; 0] | -0.035 | 0.701 | 0 [0 ; 0] | 0.074 | 0.42 | 0 [0 ; 0] | 0.101 | 0.272 |
|  | **Smoking** | 10.05 [-26.72 ; 65.28] | 0.043 | 0.641 | 7.31 [-32.68 ; 71.05] | 0.027 | 0.765 | -24.82 [-62.04 ; 48.9] | -0.076 | 0.409 |
|  | **Diabetes** | -2.28 [-46.35 ; 78.00] | -0.007 | 0.939 | 22.02 [-15.11 ; 75.39] | 0.099 | 0.279 | **64.36 [2.29 ; 164.08]** | **0.190** | **0.04** |
|  | **Statin** | 1.21 [-38.25 ; 65.89] | 0.004 | 0.962 | 6.60 [-31.47 ; 65.81] | 0.026 | 0.775 | -21.79 [-53.17 ; 30.62] | -0.087 | 0.344 |
|  | **Antihypertensive** | -22.71 [-54.08 ; 30.08] | -0.090 | 0.329 | 2.31 [-32.72 ; 55.59] | 0.010 | 0.914 | 13.43 [-38.46 ; 109.06] | 0.038 | 0.682 |
|  | **Stroke** | -8.76 [-47.52 ; 58.61] | -0.030 | 0.743 | 53.36 [-21.3 ; 198.85] | 0.116 | 0.206 | 105.08 [-15.76 ; 399.24] | 0.146 | 0.112 |
|  | **Coronary Heart Disease** | -9.61 [-64.01 ; 127.01] | -0.02 | 0.828 | 67.14 [-13.17 ; 221.74] | 0.142 | 0.123 | -44.15 [-82.63 ; 79.62] | -0.091 | 0.324 |
|  | **HDL-Cholesterol** | 16.88 [-25.49 ; 83.35] | 0.063 | 0.494 | 25.97 [-17.05 ; 91.31] | 0.100 | 0.276 | -0.51 [-38.91 ; 62.04] | -0.002 | 0.984 |
|  | **Cholesterol** | -16.71 [-33.79 ; 4.77] | -0.144 | 0.117 | 5.09 [-13.84 ; 28.19] | 0.045 | 0.621 | -9.07 [-32.87 ; 23.17] | -0.057 | 0.535 |
|  | **Diastolic** | 0.78 [-2.61 ; 4.29] | 0.041 | 0.654 | **3.58 [0.8 ; 6.44]** | **0.234** | **0.012** | -0.49 [-3.77 ; 2.92] | -0.026 | 0.775 |
|  | **Systolic** | 0.16 [-1.82 ; 2.19] | 0.015 | 0.871 | -0.56 [-1.83 ; 0.72] | -0.080 | 0.384 | 0.79 [-1.15 ; 2.77] | 0.073 | 0.423 |
| **Parieto-**  **occipital** | **Sex** | -10.34 [-49.69 ; 59.79] | -0.034 | 0.709 | 2.14 [-37.89 ; 67.96] | 0.008 | 0.933 | 10.87 [-39.37 ; 102.75] | 0.031 | 0.735 |
|  | **Age** | 3.06 [-0.62 ; 6.88] | 0.150 | 0.104 | **9.28 [5.85 ; 12.83]** | **0.503** | **<0.0001** | **3.47 [0.45 ; 6.58]** | **0.208** | **0.025** |
|  | **Years Education** | -0.72 [-6.92 ; 5.89] | -0.020 | 0.823 | 0.34 [-5.8 ; 6.87] | 0.010 | 0.915 | 5.49 [-4.14 ; 16.09] | 0.102 | 0.269 |
|  | **TIV** | 0 [0 ; 0] | 0.000 | 0.996 | 0 [0 ; 0] | 0.000 | 1 | 0 [0 ; 0] | 0.172 | 0.062 |
|  | **Smoking** | -7.81 [-40.75 ; 43.42] | -0.033 | 0.716 | -48.7 [-83.9 ; 63.46] | -0.104 | 0.256 | -24.40 [-60.92 ; 46.22] | -0.077 | 0.402 |
|  | **Diabetes** | -7.25 [-47.20 ; 62.92] | -0.024 | 0.792 | 30.04 [-16.25 ; 101.91] | 0.108 | 0.239 | 25.50 [-30.61 ; 126.97] | 0.069 | 0.449 |
|  | **Statin** | 8.75 [-36.47 ; 86.18] | 0.028 | 0.757 | -21.55 [-53.62 ; 32.71] | -0.084 | 0.362 | -22.76 [-56.21 ; 36.23] | -0.082 | 0.369 |
|  | **Antihypertensive** | -29.33 [-59.24 ; 22.54] | -0.114 | 0.214 | -19.85 [-48.88 ; 25.66] | -0.089 | 0.331 | 13.26 [-41.13 ; 117.89] | 0.035 | 0.705 |
|  | **Stroke** | -20.50 [-56.81 ; 46.34] | -0.068 | 0.457 | **443.67 [144.52 ; 1108.8]** | **0.385** | **<0.0001** | **239.66 [34.29 ; 759.08]** | **0.239** | **0.010** |
|  | **Coronary Heart Disease** | -34.39 [-73.08 ; 59.9] | -0.086 | 0.35 | 33.08 [-40.79 ; 199.11] | 0.064 | 0.486 | -3.01 [-80.68 ; 386.97] | -0.003 | 0.970 |
|  | **HDL-Cholesterol** | 0.70 [-37.71 ; 62.81] | 0.003 | 0.977 | 42.55 [-8.39 ; 121.82] | 0.145 | 0.115 | 3.84 [-41.01 ; 82.78] | 0.012 | 0.895 |
|  | **Cholesterol** | -17.08 [-37.38 ; 9.8] | -0.121 | 0.189 | -5.67 [-26.24 ; 20.62] | -0.043 | 0.639 | -10.91 [-35.14 ; 22.38] | -0.066 | 0.471 |
|  | **Diastolic BP** | 0.49 [-2.95 ; 4.06] | 0.026 | 0.779 | 2.76 [-0.4 ; 6.03] | 0.158 | 0.087 | -0.80 [-3.92 ; 2.42] | -0.046 | 0.617 |
|  | **Systolic BP** | 0.30 [-1.65 ; 2.29] | 0.028 | 0.763 | 0.22 [-1.33 ; 1.78] | 0.025 | 0.784 | 0.88 [-0.98 ; 2.79] | 0.085 | 0.352 |
